# Cerebral epidural empyema due to *Bartonella henselae*: a case report

**DOI:** 10.1186/s12879-021-06488-8

**Published:** 2021-08-06

**Authors:** Stéphanie Matta, Audrey Rousseau, Rachel Chenouard, Carole Lemarié, Matthieu Eveillard, Marie Kempf, Rafaël Mahieu, Hélène Pailhoriès

**Affiliations:** 1 Laboratoire de Bactériologie, Institut de Biologie en Santé - PBH, CHU Angers, 4 rue Larrey, 49933 Angers cedex, France; 2 Département de Pathologie Cellulaire et Tissulaire, CHU d’Angers, 4, rue Larrey, 49933 Angers, France; 3 Service de Maladies Infectieuses et Tropicales, CHU Angers, CHU, 4 rue Larrey, 49933 Angers cedex, France; 4 CRCINA, INSERM, Université de Nantes, Université d’Angers, Angers, France; 5 Laboratoire HIFIH, UPRES EA3859, SFR 4208, Université d’Angers, Angers, France

**Keywords:** *Bartonella henselae*, Cerebral epidural empyema, 16S rDNA gene sequencing, Cat Scratch Disease, Case report

## Abstract

**Background:**

Cat scratch disease frequently involves a benign, self-limited disease. Neurological forms associated with *Bartonella henselae* are uncommon, consisting mostly in neuroretinitis, encephalitis and meningitis. Cerebral epidural empyema has never described.

**Case presentation:**

An adult patient was hospitalized for isolated headaches. Magnetic Resonance Imaging (MRI) identified typical features of cerebral epidural empyema. The diagnosis of *B. henselae* was performed incidentally by 16S rDNA gene sequencing on the abscess fluid, and confirmed by specific qPCR. We report here the first case, to our knowledge, of cerebral epidural empyema associated with *B. henselae.* Further follow-up visits allowed identifying frequent cat scratches on the scalp as the presumptive source of infection.

**Conclusions:**

This case report alerts about such atypical clinical presentation, which requires an extensive clinical investigation. It also emphasizes on the usefulness of additional molecular diagnosis techniques in such CNS infection cases.

## Background

*Bartonella henselae* is a bacterium responsible for cat scratch disease (CSD), a zoonotic infectious disease usually transmitted to human by bites or scratches of domestic cat, its natural reservoir. Atypical clinical course in CSD occurs in a minority of cases (5–14% of CSD) [1]. Among these atypical CSD, neurological manifestations associated with *B. henselae* are scarce, consisting mostly in neuroretinitis, meningoencephalitis and myelitis [2]. To our knowledge, cerebral epidural empyema has never been described in the literature. We report here the case of a cerebral epidural empyema due to *B. henselae* diagnosed incidentally by 16S rDNA gene sequencing on the abscess fluid.

## Case presentation

An adult patient visited his general practitioner for a five-day history of high fever, diffuse abdominal pain and myalgia. Symptoms first disappeared and relapsed a few days later with the onset of gradual worsening headaches. A corticosteroid therapy was initiated considering sinusitis as a possible cause of the headaches, resulting in a complete resolution of all symptoms. After one week of treatment interruption, the headaches resumed and prompted the patient's admission to the hospital. He had no past medical history except for severe acne, tonsillectomy and surgery for a sinus polyp. No medication was reported at the time of admission. The patient worked as a welder and had a 5-year-old son. No exposure to animals was reported except for his cat.

**Fig. 1:**
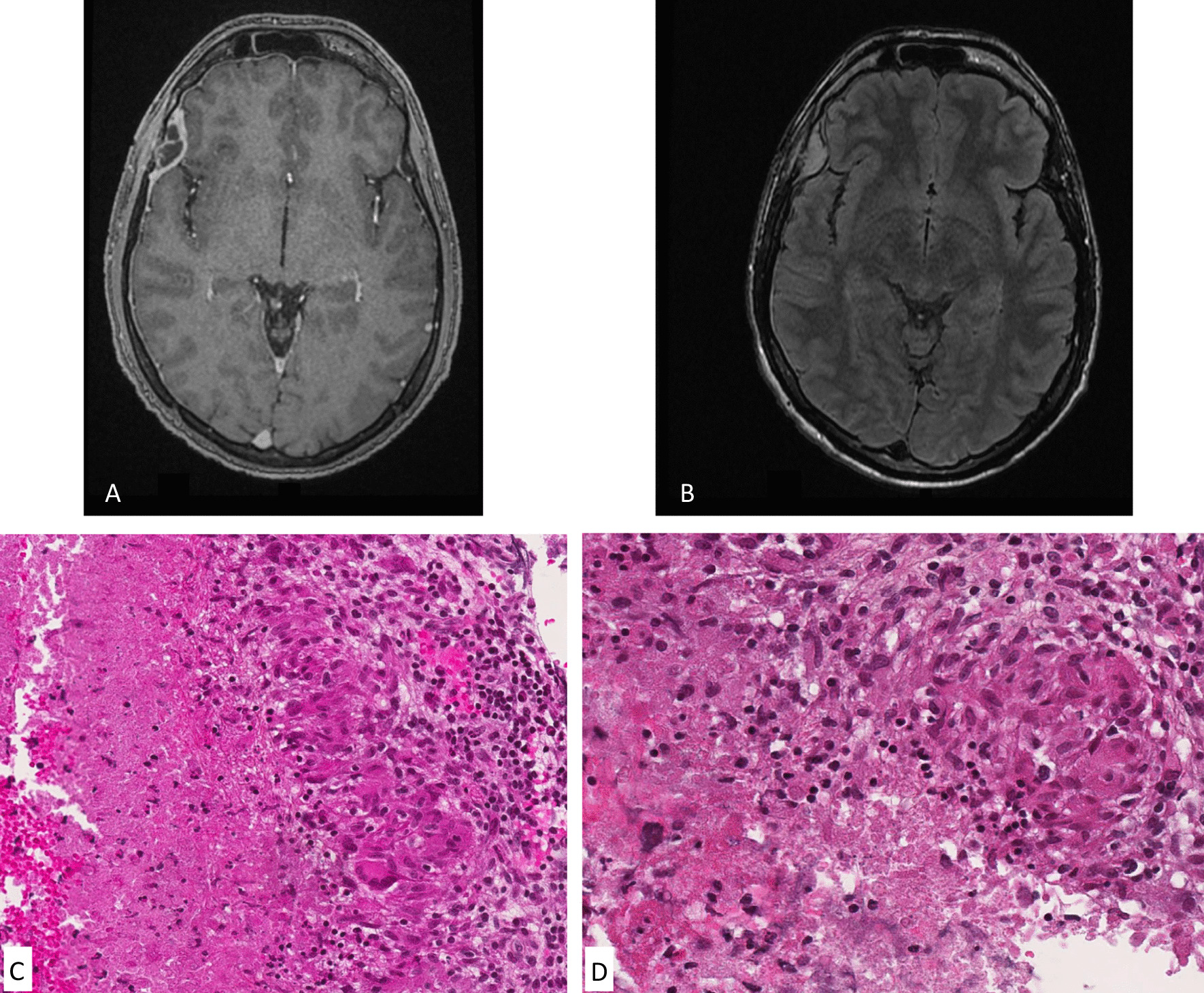
**A** MRI showing a gadolinium enhancing extra-dural lesion **B** MRI with FLAIR hyperintensities **C** Granulomatous inflammation (on the right) with epitheliod histiocytes and multinucleated giant cells. On the left, presence of suppurated necrosis. (hematoxylin phloxine saffron staining (HPS), 200×). **D** Epithelioid and giant cell granuloma (on the right) associated with suppurated necrosis (HPS, 200×)

On hospital admission, clinical examination revealed a body temperature of 36.4 °C. The patient reported isolated headaches. A cerebral computed tomography scan (CT) identified an epidural collection with contrast enhancement in the right frontoparietal area. The cerebral Magnetic Resonance Imaging (MRI) showed findings typical of a cerebral epidural empyema with FLAIR hyperintensities with gadolinium enhancing lesions (Fig. 1A, B). The thoraco-abdomino-pelvic CT was normal. The cerebrospinal fluid (CSF) analysis detected 30 WBCs/mm3 including 85% of lymphocytes, with normal protein and glucose levels. The bacteriological cultures of the CSF were sterile after a 48-h incubation on sheep blood and chocolate agar plates in air atmosphere with 5% CO_2_ as well as on Brain–Heart enrichment broth.

A surgical drainage of the epidural collection was performed, with bacteriological and pathological analyses. The direct examination with Gram staining was negative. The bacteriological cultures remained sterile after 7 days of incubation on sheep blood agar in aerobic and anaerobic conditions, on chocolate agar in air with 5% CO_2_ and in Schaedler and Brain–Heart enrichment broths.

Post-operative examination was normal with complete resolution of the headaches. The serum biochemical parameters were within reference range, the serum protein electrophoresis showed a restricted heterogeneity of gamma globulins, corresponding to a slightly irregular distribution of gamma globulins. No coagulation disorder was recorded and a normal level of thyroid stimulating hormone (TSH) was observed. The C-reactive protein level was normal (4 mg/L, reference value ≤ 6 mg/L). The serologies for human immunodeficiency virus, hepatitis B virus, hepatitis C virus, toxocariasis and hydatidosis were negative. Microscopic examination of the epidural collection by the pathologist (AR) showed a granulomatous inflammation associated with necrosis and granular debris; neutrophils were recognizable. Grocott and Ziehl–Neelsen stainings were negative. There were no signs of malignancy (Fig. 1C, D). Meanwhile, 16S rDNA gene sequencing was performed on the abscess fluid with Sanger sequencing protocol, using the universal primer pair fD1 (5'-AGAGTTTGATCCTGGCTCAG-3') and rP2 (5'-ACGGCTACCTTGTTACGACTT-3') [3]. The result was analyzed using Basic Local Alignment Tool (BLAST) program through the National Center for Biotechnology Information (NCBI) server (https://blast.ncbi.nlm.nih.gov/Blast.cgi). An identification of *B. henselae* was obtained. This result was confirmed by an in-house PCR targeting the rRNA 23S *B. henselae* gene (BH5 5’-GAGGCCCCTACCTCTGAAAGA-3’ and BH6 5’-TCAAAGCCCACGGTGGA-3’). During the follow-up of the patient, it turned out that his cat made frequent scratches on his scalp.

A 1 month course of doxycycline (100 mg bid) was prescribed with no relapse during a two-year follow-up.

## Discussion and conclusions

To the best of our knowledge, we report herein the first case of cerebral epidural empyema due to *B. henselae* described in the literature. This clinical description raises the question about the pathophysiology of Cat Scratch Disease (CSD) in this patient. Following inoculation of *B. henselae*, most patients with CSD present a benign self-limited infection. CSD is one of the leading causes of lymphadenopathy in children and adolescents but the exact interaction between the host immune system and the bacterium remains unknown. By invading endothelial cells with an increased production of vascular endothelial growth factor, *B. henselae* has also been associated with systemic diseases like endocarditis, hepatosplenic abscesses, retinopathy, uveitis, peliosis hepatitis, osteomyelitis, splenomegaly, pneumonia and glomerulonephritis [2]. Some of these manifestations have been mostly described in patients presenting an immunocompromised status like bacillary angiomatosis (BA) but the pathogenesis of each form of CSD remains poorly understood while documented BA in immunocompetent adults has also been reported [4, 5]. Most neurological manifestations consist of neuroretinitis, encephalitis and meningitis. Peripheral neuropathy and acute hemiplegia have also been described during CSD. Diagnosis of CSD-associated encephalopathy may be challenging as normal MRI have been described despite the existence of neurological deficits [6]. Other manifestations include status epilepticus [7], cerebral vasculitis, with secondary cerebral infarction and necrosis [8], or brainstem encephalopathy with basal ganglia impairment [9]. Spinal epidural abscesses due to *B. henselae* have also been detailed in the literature [10–12]. In these cases, the spinal abscesses were all described in pediatric context (children from 5 to 10 years old) and associated with vertebral osteomyelitis. However, cerebral epidural empyema associated with *B. henselae* has never been reported in the literature. We suggest that this uncommon manifestation may reflect the atypical portal of entry of *B. henselae* in this patient with the cerebral epidural empyema corresponding to a localized manifestation of CSD. During a follow-up interview, the patient acknowledged that his cat had scratched his head on several occasions. Moreover, cerebral abscesses or empyema may be associated with local infection (sinusitis, acute medium otitis) or may appear in the post-operative setting. Haematogenous spread can be associated with brain abscesses and may complicate the clinical course of CSD endocarditis [13]. However, this source of contamination is unlikely in this case, the most likely transmission route being the scratches on the scalp. The emissary veins of the scalp could thus have played a role in the dissemination of the pathogen, providing a connection between the extracranial tissues and the intracranial transverse sinus, and possibly the epidural space. The initial prescription of corticosteroids without antibiotics could also contribute to the intracranial extension of the infection. The development of the granulomatous lesion in the epidural space was thus associated with the multiplication of *B. henselae*.

Because *B. henselae* is a fastidious bacterium that requires specific laboratory conditions, conventional growth techniques performed on cerebral samples may lack sensitivity for this pathogen. Overall, 7 to 53% of cultures of cerebral suppuration remain negative with conventional techniques. Because streptococci and anaerobes are the most commonly isolated microorganisms from empyema material and because most negative cultures are related to previous antibiotic treatment [14], the use of complex additional techniques may have been considered as futile. Recently, the causative bacteria involved in central nervous system (CNS) infections have been studied through a systematic comparison between 16S-rDNA-based next-generation sequencing and conventional techniques [15]. Molecular assays were able to identify a larger number of bacterial taxa compared to culture with the identification of several species infrequently recorded in these infections. Therefore, the absence of identification of infective microorganisms in a CNS infection requires an extensive investigation and the use of additional molecular-based techniques.

Most patients with CSD lymphadenitis experience a spontaneous resolution of their symptoms without specific antibiotic therapy. The fact that the antibiotic treatment may shorten the duration of symptoms or decrease the risk of systemic disease is debated in the literature [16, 17]. However, in a large cohort of 268 patients with CSD lymphadenitis, antibiotic treatment was associated with a median duration of symptoms of 2.8 weeks compared with 14.5 weeks in patients without antibiotherapy [17]. Limited data are available for more severe forms of the disease but a combination of active agents against *B. henselae* is commonly used with a favorable outcome after a 4-week regimen [18]. A combination of doxycycline and rifampicin [2, 7, 9, 19] or azithromycin and rifampicin has been proposed for the management of CNS infections associated with *B. henselae*, in particular for encephalitis [7]. Because of their specific pathophysiology, intracranial epidural abscesses may be easier to treat than cerebral abscesses or encephalitis with the specific impact of surgical drainage. In our case, the evolution was favorable with surgical drainage and a one-month course of doxycycline.

This case report describes a first case of *B. henselae* cerebral epidural empyema. This emphasizes the potential unusual clinical presentation of *B. henselae* infection, which has been treated here by surgical drainage and appropriate antibiotic treatment. Furthermore, in CNS infections with negative microbiological culture results, the use of additional molecular techniques seems essential to the microbiological diagnosis.

## Data Availability

All data generated or analysed during this study are included in this published article.

## Abbreviations

BA: Bacillary angiomatosis
BLAST: Basic Local Alignment Tool
CNS: Central nervous system
CSD: Cat scratch disease
CSF: Cerebrospinal fluid
MRI: Magnetic resonance imaging
NCBI: National Center for Biotechnology Information
